# TRPM7 silencing attenuates Mg^2+^ influx in cardiac myoblasts, H9c2 cells

**DOI:** 10.1186/s12576-020-00772-z

**Published:** 2020-10-07

**Authors:** Michiko Tashiro, Masato Konishi, Ryo Kobayashi, Hana Inoue, Utako Yokoyama

**Affiliations:** 1 Department of Physiology, Tokyo Medical University, 6-1-1 Shinjuku, Shinjuku-ku, Tokyo, 160-8402 Japan; 2 Department of Microbiology, Tokyo Medical University, Tokyo, 160-8402 Japan

**Keywords:** Magnesium, TRPM7, Cardiac myoblast, H9c2, Mag-fura-2

## Abstract

TRPM7, a member of the melastatin subfamily of transient receptor potential channels, is suggested to be a potential candidate for a physiological Mg^2+^ channel. However, there is no direct evidence of Mg^2+^ permeation through endogenous TRPM7. To determine the physiological roles of TRPM7 in intracellular Mg^2+^ homeostasis, we measured the cytoplasmic free Mg^2+^ concentration ([Mg^2+^]_i_) in TRPM7-silenced H9c2 cells. [Mg^2+^]_i_ was measured in a cluster of 8–10 cells using the fluorescent indicator, furaptra. TRPM7 silencing did not change [Mg^2+^]_i_ in Ca^2+^-free Tyrode’s solution containing 1 mM Mg^2+^. Increasing the extracellular Mg^2+^ to 92.5 mM raised [Mg^2+^]_i_ in control cells (1.56 ± 0.19 mM) at 30 min, while this effect was significantly attenuated in TRPM7-silenced cells (1.12 ± 0.07 mM). The Mg^2+^ efflux driven by Na^+^ gradient was unaffected by TRPM7 silencing. These results suggest that TRPM7 regulates the rate of Mg^2+^ influx in H9c2 cells, although cytoplasmic Mg^2+^ homeostasis at basal conditions is unaffected by TRPM7 silencing.

## Background

The importance of intracellular Mg^2+^ has been widely recognized. Mg^2+^ is essential for protein synthesis, the regulation of ion channels, and as a co-factor in over 600 enzymatic reactions, many of which affect cellular functions and viability [1]. Thus, cytoplasmic free Mg^2+^ concentration ([Mg^2+^]_i_) should be kept in physiological range, but the molecules responsible for Mg^2+^ influx pathway remain to be identified. In cardiac myocytes, several candidates, such as transient receptor potential melastatin subfamily member 7 (TRPM7, non-selective cation channel) and magnesium transporter 1 (MagT1, Mg^2+^-selective channel), have been proposed [2, 3]. The properties of these channels have been investigated by heterologous overexpression systems [4, 5], and the data driven by these types of studies may not always be associated with the physiological functions of endogenous Mg^2+^-related channels.

The low rate of Mg^2+^ influx under physiological conditions makes it difficult to identify endogenous Mg^2+^ channels. We previously quantified Mg^2+^ entry in rat ventricular myocytes, and found that the rate of Mg^2+^ influx was altered by TRPM7 modulators in a concentration-dependent manner with EC50 (half-maximal effective concentration) values comparable with those reported for the TRPM7 channel activities [6, 7]. To extend these pharmacological studies suggesting that TRPM7 functions as a physiological pathway of Mg^2+^ influx in native cardiac myocytes, we investigated the rate of Mg^2+^ transport of the cardiac myoblasts (H9c2) transfected with TRPM7-targeted shRNA. Some of the results have been reported in abstract form [8].

## Methods

### Cells

H9c2 (2-1) rat-cardiac myoblast cells from ECACC were cultured on glass-bottom dishes (D11130H, Matsunami-Glass, Osaka) with DMEM including 1.8 mM CaCl_2_ and 0.8 mM MgSO_4_ (D6429; Sigma-Aldrich, St. Louis, MO) supplemented with 10% FBS, 100 U/mL penicillin G and 0.1 mg/mL streptomycin, in the 37 °C, 5% CO_2_ humidified incubator.

### RNA interference

Cells were transfected by lipofectamine 2000 (Thermo Fisher Scientific, Waltham, MA) with plasmid DNA encoding shRNA of rat *Trpm7* (shTRPM7) or non-targeting shRNA (shControl), and GFP gene as a marker of introduction (Sure Silencing shRNA plasmids purchased from Qiagen, Hilden, Germany). The shRNA-*Trpm7* target sequence was 5′-AGCGTTTGACCAGCTTATCCTTA-3 while that for the non-targeting shRNA was 5′-GGAATCTCATTCGATGCATAC -3′. Three days after transfection, cells were used for experiments.

### Cell viability and transfection efficiency

Cells (1 × 10^5^/mL/well) were plated onto a 12-well plate with DMEM (10% FBS) one day before transfection. On several days after transfection, the cells were dissociated with TrypLE™ Express Enzyme (Gibco, Thermo Fisher Scientific), and counted with the hemocytometer to assess viability using 0.4% Trypan Blue Solution (Gibco). We also counted GFP-positive cells using the same chamber through fluorescence microscopy to check transfection efficiency.

### Quantitative real-time PCR

Total RNA was isolated from H9c2 using the SV total RNA isolation system (Promega, Madison, WI) and converted to cDNA using the high-capacity reverse transcription kit (Applied Biosystems, Foster City, CA) according to manufactures’ protocols. The expression of mRNA of TRPM7 was determined by quantitative real-time polymerase chain reaction (Applied Biosystems 7500 Real-time PCR system) using TaqMan probe sets (Thermo Fisher Scientific) for rat TRPM7 (Rn00586779_m1) relative to rat glyceraldehyde-3-phosphate dehydrogenase (GAPDH, Rn99999916_s1).

### Immunoblotting

The cells were homogenized by sonication in lysis buffer (150 mM Na_2_CO_3_, 1 mM EDTA-Na). These samples were mixed with sodium dodecyl sulfate (SDS) buffer (0.5 M Tris–HCl, SDS, glycerol, BPB, and β-mercaptoethanol) and boiled at 100℃ for 5 min. Proteins were separated by SDS-PAGE (6% for TRPM7, 10% for GAPDH) and electrically transferred onto a polyvinylidene difluoride membrane. The membrane was incubated with an anti-TRPM7 antibody (ab109438; Abcam Biochemicals, Bristol, UK) or an anti-GAPDH antibody (SC-25778; Santa Cruz Biotechnology, Dallas, TX, USA) followed by peroxidase-conjugated anti-rabbit IgG antibody (CST#7074S; Cell Signaling Technology, Danvers, MA, USA). The positive bands were visualized using the SuperSignal™ West Dura Extended Duration Substrate (Thermo Fisher Scientific) and the enhanced chemiluminescence system of Chemi DOC™, and then analyzed with software, imageLab4.1™ (Bio-Rad Laboratories, Hercules, CA).

### Measurements and analysis of furaptra signals

The instruments and procedures for the measurements of furaptra (mag-fura-2)-fluorescence signals from cells have been described previously [7, 9]. Briefly, H9c2 cells on the glass-bottom culture dish was placed on the stage of an inverted microscope (TE300; Nikon, Tokyo) and was superfused with Ca^2+^-free Tyrode’s solution (see Solutions). The intracellular fluorescence was alternately excited with 350 nm and 382 nm light beams, and the fluorescence at 500 nm (25 nm bandwidth) was detected from the cluster of 8–10 cells including 4–5 cells labeled GFP. The area of illumination by excitation light was limited to the cluster size with an aperture diaphragm. After measurement of the background fluorescence from the cluster, cells were loaded with 5 μM furaptra AM (Invitrogen, Life Technologies, Carlsbad, CA) by incubation in Ca^2+^-free Tyrode’s solution for 14 min at room temperature, and the acetoxy methyl (AM) ester was washed out with Ca^2+^-free Tyrode’s solution for 10 min. Subsequent fluorescence measurements were carried out at 25 °C under Ca^2+^-free conditions to minimize possible cell damage and interference in the furaptra fluorescence caused by Ca^2+^ overloading of the cells.

The ratio of furaptra fluorescence intensities excited at 382 and 350 nm after background subtraction, *R* = F(382)/F(350), was converted to [Mg^2+^]_i_ according to the equation:

1 $$ [{\text{Mg}}^{2 + } ]_{{\text{i}}} = K_{D} \cdot \frac{{R - R_{\min } }}{{R_{\max } - R}}, $$

where *K*_*D*_ is the dissociation constant, and *R*_min_ and *R*_max_ are *R* values at zero [Mg^2+^] and saturating [Mg^2+^], respectively. We used the parameter values previously estimated in rat ventricular myocytes (*K*_*D*_ = 5.30 mM, *R*_max_ = 0.223 [10] and *R*_min_ = 0.967 [7]).

Influx of Ni^2+^ was monitored by fluorescence quenching of intracellular furaptra as previously described [6]. We measured the decrease in furaptra fluorescence intensity excited at 350 nm (an isosbestic wavelength for Mg^2+^) induced by substitution of 1 mM Ni^2+^ for Mg^2+^ of Ca^2+^-free Tyrode’s solution at 25 °C.

### Analysis of the rate of Mg^2+^ influx and efflux

After furaptra loading, the cells were initially perfused with high-Mg^2+^solution for 30 min, then were reperfused with Ca^2+^-free Tyrode’s solution for 30 min. [Mg^2+^]_i_ was measured at ~ 5-min intervals during the perfusion. Because the [Mg^2+^]_i_ rise is likely caused by the influx of Mg^2+^, we analyzed the rate of increase in [Mg^2+^]_i_ as Mg^2+^ influx rate by Mg^2+^-loading.

The rate of Na^+^-dependent Mg^2+^ efflux was analyzed as described previously [11]. In brief, the cells were incubated in Mg^2+^-loading solution (see Solutions) for varying periods up to 2 h, until [Mg^2+^]_i_ was elevated to 1.5 mM or higher. Introduction of extracellular Na^+^ by perfusion of Ca^2+^-free Tyrode’s solution induced the Na^+^-dependent Mg^2+^ efflux. The initial rate of decrease in [Mg^2+^]_i_ was estimated by linear regression of data points spanning for 120 s (30–150 s after solution exchange).

### Solutions

Ca^2+^-free Tyrode’s solution contained (mM): 135 NaCl, 5.4 KCl, 1.0 MgCl_2_, 0.33 NaH_2_PO_4_, 5.0 glucose, 10 HEPES and 0.1 K_2_EGTA (pH 7.40 at 25 °C by NaOH). High-Mg^2+^solution contained 68.5 mM MgCl_2_ and 24 mM MgMs_2_ in place of 135 mM NaCl and 1.0 mM MgCl_2_ of Ca^2+^-free Tyrode’s solution. Mg^2+^-loading solution contained 24 mM [Mg^2+^], which was prepared by substitution of 135 mM NaCl of Ca^2+^-free Tyrode’s solution with 101 mM NMDG-Cl (N-methyl-D-glucamine titrated HCl), 19.6 mM MgCl_2_ and 6.0 mM MgMs_2_.

### Statistics

Linear and nonlinear least-squares curve fitting was performed with the program Origin (Ver. 9.1, Origin Lab, Northampton, MA, USA). Statistical values are expressed as the mean ± SE. Differences between groups were analyzed by Student’s *t*-test or two-way repeated measure ANOVA with the significance level set at *p* < 0.05 (Ver. 26, IBM SPSS statistics).

## Results

**Fig. 1:**
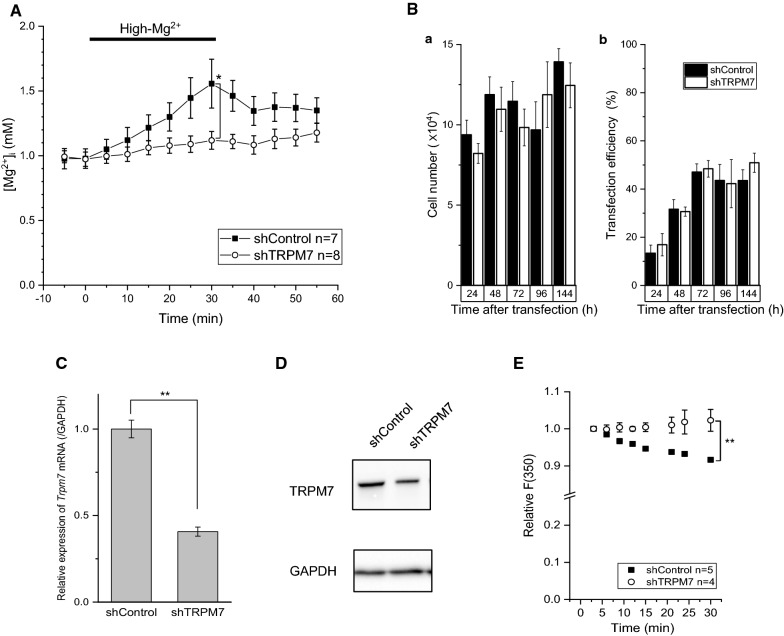
TRPM7 silencing attenuates the increment in [Mg^2+^]_i_ of H9c2 cells by incubation in high-Mg^2+^ solution. **A** Each symbol represents mean ± SE of [Mg^2+^]_i_ obtained from shControl cells or shTRPM7 cells. Cells were perfused with Ca^2+^-free Tyrode’s solution (1 mM Mg^2+^), except during the period of perfusion with high-Mg^2+^ (95 mM) solution indicated by the line. **B** Total number of the cells (**a**) and transfection efficiency (**b**) are plotted as a function of time after transfection with non-targeting (*black* columns) or TRPM7 (*white* columns) shRNA. Each column shows mean ± SE of the data from 3 different experiments. There was no statistical difference of cell number (shControl vs shTRPM7) in any time between 24 and 144 h. **C** Quantitative RT-PCR analysis of TRPM7 mRNA expression relative to that of GAPDH. Each column shows mean ± SE of the data obtained from, respectively, shControl cells (1.0 ± 0.051, *n* = 4) or shTRPM7 cells (0.407 ± 0.026, *n* = 4). **D** Western blot analysis of protein expression of TRPM7 in shControl and shTRPM7 cells. **E** Relative fluorescence intensities of furaptra from shControl or shTRPM7 cells are plotted against time after administration of Ni^2+^. *0.01 ≤ *p* < 0.05, **p < 0.01

In Ca^2+^-free Tyrode’s solution, [Mg^2+^]_i_ of TRPM7-silenced H9c2 cells (shTRPM7 cells) was 0.99 ± 0.05 mM, which was similar to that of shControl cells (0.98 ± 0.08 mM) (Fig. 1A). The levels of [Mg^2+^]_i_ in shControl cells were raised gradually by perfusion with high-Mg^2+^ solution (92.5 mM), and then partially recovered by reperfusion of Ca^2+^-free Tyrode’s solution containing 1 mM Mg^2+^. On the other hand, the increment in [Mg^2+^]_i_ (1.12 ± 0.07 mM) in shTRPM7 cells was significantly smaller than that of shControl cells (1.56 ± 0.19 mM, *p* = 0.034) (Fig. 1A).

We confirmed whether TRPM7 was silenced in the shTRPM7 cells compared with the shControl cells. Figure 1B shows that the number of cells was not changed at least up to 144 h after transfection of both of plasmids (shControl 1.15 ± 0.12 × 10^5^ and shTRPM7 0.98 ± 0.12 × 10^5^ at 72 h after transfection, *n* = 3, *p* = 0.38). There was no significant difference of cell viability between shControl cells and shTRPM7 up to 144 h (91.8 ± 2.1 and 89.6 ± 3.8%, respectively, at 72 h after transfection, *n* = 3, *p* = 0.63).

Since the transfection efficiency reached about 50% in 72 h (Fig. 1B), we used the cells for experiments three days after transfection. After RNA interference with *Trpm7* shRNA, the relative expression level of *Trpm7* mRNA was significantly reduced to 41 ± 3% in shTRPM7 cells compared with shControl cells (Fig. 1C). The expression level of TRPM7 protein was decreased to 58.7% (Fig. 1D**)**. To further confirm the efficiency of TRPM7 silencing, we estimated TRPM7 channel activity using Ni^2+^ influx monitored by quenching of intracellular furaptra. Figure 1E shows that Ni^2+^ quenching of fluorescence in shTRPM7 cells was significantly smaller than that of shControl cells (*p* = 0.009, two-way repeated measures ANOVA), suggesting impairment of TRPM7 channel activity on the plasma membrane of shTRPM7 cells.

**Fig. 2:**
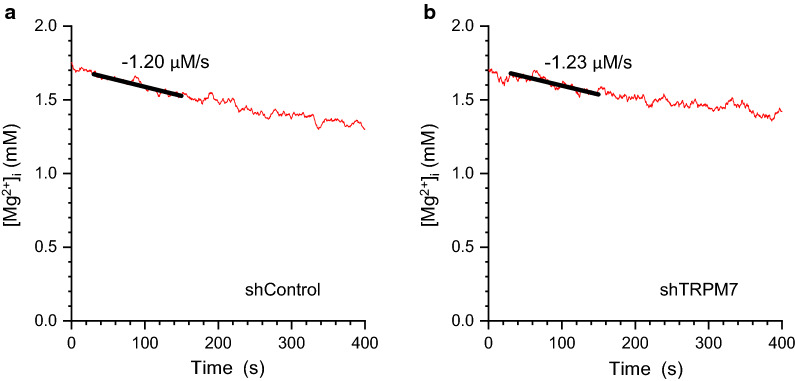
Examples for analysis of the Mg^2+^-efflux rate in shControl (**a**) and shTRPM7 (**b**) cells. In **a** and **b**, the Na^+^-dependent Mg^2+^ efflux was induced by the addition of 140 mM Na^+^ to the Mg^2+^-loaded cells at time 0. Solid lines were drawn by linear-fitting to unsmoothed data points spanning 120 s

We further investigated the effect of TRPM7 silencing on Mg^2+^ efflux, as shown in Fig. 2. Excessive Mg^2+^ in rat ventricular myocytes is mainly extruded with a Na^+^-gradient-dependent Mg^2+^-efflux system [11]. The Na^+^-dependent Mg^2+^ efflux was induced by introduction of extracellular Na^+^ to the Mg^2+^-loaded cells (see Methods). The initial rate of decrease in [Mg^2+^]_i_ was, on average, 1.02 ± 0.06 μM/s in shTRPM7 cells (*n* = 5) and 1.02 ± 0.21 μM/s in shControl cells (*n* = 5); these values were not significantly different.

## Discussion

TRPM7 channel has been characterized in heterologous overexpression systems, as Mg^2+^-permeable channel which is inactivated by intercellular free Mg^2+^, and its Mg^2+^ sensitivity is regulated by in vivo factors such as nucleotides and oxidative stress [12, 13]. Regarding cardiac myocytes, a TRPM7-like current was demonstrated in rat, guinea pig and pig ventricular myocytes [14, 15], and human atrial myocytes [3, 16]. Sah et al. described the importance of TRPM7 in maintaining cardiac automaticity in the sinoatrial node [17]. They also reported that TRPM7 was critical for cardiogenesis based on the results of cardiac myocyte-targeted knockout mice [18].

The present study focuses on the involvement of TRPM7 as an Mg^2+^ influx pathway in cardiac myocytes. Since we have studied on Mg^2+^-regulation mechanisms in adult rat ventricular myocytes [2, 6, 7], we initially tried to knockdown TRPM7 in primary cultures of these cells using shRNA (+ GFP) transfection with adenovirus vector. However, it was not possible to detect a significant decrease in TRPM7-like currents in shTRPM7 (GFP positive) ventricular myocytes, probably because of protein turnover of endogenous TRPM7. Since the H9c2 cell line, myoblasts derived from rat heart, have been widely used as in vitro model of cardiac myocytes [19, 20], we used H9c2 cells to investigate the roles of TRPM7 in Mg^2+^ regulation.

The rate of increase in [Mg^2+^]_i_ by inflow of extracellular Mg^2+^ is very slow [21]. A long-time soaking in high-Mg^+^ solutions caused damage and morphological changes in H9c2 cells. We therefore used extremely high (92.5 mM)-Mg^2+^ solution to observe Mg^2+^ influx in a short period (30 min). TRPM7 silencing significantly decreases the rate of Mg^2+^ influx of the cells at high [Mg^2+^]_o_, although basal [Mg^2+^]_i_ appears to still be maintained at normal [Mg^2+^]_o._ TRPM7 silencing had little effect on the activity of the Na^+^-dependent Mg^2+^ efflux. The results indicate that TRPM7 serves as a physiological Mg^2+^ influx pathway, but it might not be the sole key player to maintain basal [Mg^2+^]_i_ in H9c2 cells.

The necessity of TRPM7 for cellular Mg^2+^ homeostasis in DT40 (chicken B lymphocytes) has been described by Schmitz et al. [22]. The authors also reported that deletion of TRPM7 in DT40-upregulated expression of MagT1 compensates for the Mg^2+^ deficiency [23]. In contrast, Jin and coworkers reported that tissue-specific deletion of TRPM7 in T lymphocytes of mice [24], and that in neural stem cells [25] does not alter total Mg contents of these cells. Castiglioni et al. reported that TRPM7/MagT1 co-silencing does not affect the intracellular total Mg content in human mesenchymal stem cells [26]. Maintenance of total Mg content even in the absence of TRPM7 [24–26] seems in line with little change in basal [Mg^2+^]_i_ with TRPM7 silencing found in the present study. Physiological roles of TRPM7 should not be denied, as reduction of Mg^2+^ influx via TRPM7 might be compensated by other channels/transporters with upregulated expression levels, particularly in immature cell types. Thus, Mg^2+^ homeostasis is likely regulated by TRPM7 in cooperation with other channels/transporters, which varies in cell types.

Although the TRPM7 gene silencing was slightly less than half in this study, it suppressed the Mg^2+^ influx induced by extracellular high Mg^2+^. To the best of our knowledge, this is the first report demonstrating that extracellular Mg^2+^ passes through endogenous TRPM7. This study also indicates that TRPM7 silencing inhibits the relatively rapid influx of Mg^2+^ driven by large concentration gradient of the ion across the cell membrane. It is then tempting to speculate that TRPM7 may exert a prominent role when [Mg^2+^]_i_ falls rapidly. In cardiac myocytes, total Mg content or [Mg^2+^]_i_ is known to markedly decrease under pathological conditions, such as hypoxia–reoxygenation [27] and heart failure [28]. Low [Mg^2+^]_i_ should activate TRPM7, and the channel may provide a rapid supply of Mg^2+^ from the extracellular space for recovery of [Mg^2+^]_i_.

## Conclusions

Our results suggest that TRPM7 appreciably participates in Mg^2+^ influx, but it might not be indispensable for Mg^2+^ homeostasis in H9c2 cells. It seems reasonable to assume that Mg^2+^ homeostasis is concertedly regulated by several Mg^2+^-permeant channel/transporters including TRPM7.

## Data Availability

The datasets used and/or analyzed during the current study are available from the corresponding author on reasonable request.
